# The Anatomist's Vade Mecum

**Published:** 1840-10

**Authors:** 


Art. II.—*The Anatomist's Vade Mecum: a system of Human Anatomy.* By W. J. Erasmus Wilson. *With upwards of* 150 *Illustrations,* by Boys.—*London,* 1840. 8vo, pp. 551.

This is probably the prettiest medical book ever published, and we believe its intrinsic merits are in keeping with its exterior advantages. By means of a small but most distinct type, set off by the smoothness and whiteness of the paper, the author has contrived to compress into one small volume a complete system of descriptive anatomy, "recording" as is justly stated in the preface, "in a clear, precise, and perspicuous style, every important detail of human structure, and the most modern and valuable discoveries and researches in the science of anatomy." It is only by perusal that one can be made to believe that in a space so small, the details can be given fully and completely. This, however, is the case; and when we find that all the verbal descriptions are again presented to the mind, through the eye, by the innumerable woodcuts so profusely scattered throughout the volume, we are compelled to admit that the *Vade Mecum* of Mr. Wilson is not only the best and most beautiful of the works of its class, but is the very *beau ideal* of what such a work for the student should be. We do not pretend to have read this volume, further than to satisfy ourselves as to its literary character, by dipping into its pages here and there; but we have examined it sufficiently to satisfy us that it may be recommended to the student as no less distinguished by its accuracy and clearness of description, than by its typographical elegance. The woodcuts are exquisite.

